# Richard Semon (1859–1918): Expeditionen, Engramme und Epigenetik

**DOI:** 10.1007/s40211-022-00454-9

**Published:** 2023-01-24

**Authors:** Hans Förstl

**Affiliations:** Institut für Geschichte und Ethik der Medizin, TUM, Ismaningerstr. 22, 81675 München, Deutschland

**Keywords:** Alzheimer-Angst, Engramm, Epigenetik, Ernst Haeckel, Gedächtnis, Paul Kammerer, Suizid, Engram, Epigenetics, Ernst Haeckel-memory, Memory loss, Paul Kammerer, Suicide

## Abstract

Richard Semon (1859–1918) war Schüler von Ernst Haeckel und beschäftigte sich als Zoologe zunächst mit der Entwicklung von Seegurken, Seesternen, Hühnern und Lungenfischen, die er unter anderem am Mittelmeer und in Australien sammelte. Nach Deutschland zurückgekehrt musst er aus privaten Gründen Jena und sein universitäres Umfeld verlassen, liess sich in München nieder, wo er sich den philosophischen Aspekten der Biologie widmete, Werke über sein Gedächtniskonzept der „Mneme“ verfasste (1904) und über die Vererbung erworbener Eigenschaften nachdachte (1912). Seine Vorstellungen von Gedächtnis gingen weit über Gehirn und Individuum hinaus. Enttäuscht über eine zu geringe wissenschaftliche Anerkennung, verzweifelt nach dem Tod seiner Frau, verunsichert durch die politische Situation am Ende des ersten Weltkriegs und vor allem von einem befürchteten Gedächtnisverlust des Gedächtnisforschers, nahm er sich das Leben. Sein wichtigster Gewährsmann, der Wiener Experimentalbiologe Paul Kammerer (1880–1926), erschoss sich acht Jahre später als Zweifel an der Vererbung erworbener Eigenschaften seiner Salamander und Geburtshelferkröten auftauchten. Neuropsychiatrisch relevante Fragen nach Epigenetik, der Natur des Gedächtnisses, nach Depression und der Furcht dem Nachlassen seiner geistigen Leistungsfähigkeit, den Auswirkungen privater Umstände auf wissenschaftliche Karrieren, nach wissenschaftlichen Irrtümern und fraglichen Fälschungen, bis zum Suizid eines Wissenschaftlers finden sich verdichtet im Leben und Tod Richard Semons.

## Abstract (en)

Richard Semon (1859–1918) was a student of Ernst Haeckel and began his career as a zoologist with work on sea urchins, starfish, chicken and lung fish, which he collected at the Mediterranean Sea and in Australia. After his return to Germany he was forced to leave Jena and the university due to private reasons, and settled in Munich, where Semon devoted most of his time to the more philosophical aspects of biology, developed the theory of “mneme” (1904), which he extended towards the inheritance of acquired characteristics (1912). Semon’s concept of memory reached far beyond the brain and the individual person. In 1918 he took his life, despondent because of a surmised lack of scientific appreciation, the death of his beloved wife, the political turmoil at the end of WWI, and his—the memory researcher’s—suspected loss of memory. Eight years later, the experimental biologist Paul Kammerer (1880–1926) from Vienna, Semon’s must trusted source for the inheritance of acquired characteristics, also shot himself. Serious doubts increasingly overshadowed his work on salamanders and midwife toads. Epigenetics, the nature of memory, the fear of cognitive impairment, depression, the impact of private and political matters on scientific work, suspected scientific errors, fraud and a scientists’ suicides are condensed in Semon’s life and death.

## Einleitung

Mnesis, das Gedächtnis, wurde von Platon (ca. 428 bis 348 v. Chr.) mit Eindrücken auf einer Wachstafel verglichen und als Ana-Mnesis verstand er den Akt der Erinnerung. Die moderne Gedächtnisforschung versucht diese Spuren mit molekularen und bildgebenden Verfahren sichtbar zu machen. Dabei wird immer wieder Richard Semon zitiert, der in seinem Werk Mneme die Begriffe Engramm und Ekphorie für das Einprägen und Hervorholen von Gedächtnisinhalten einführte (z. B. [29, 40, 45]). Überdies gewinnen Semons prinzipielle Überlegungen zur Vererbung erworbener Eigenschaften durch die moderne Epigenetik an Aktualität. Auch aus nervenärztlicher Sicht verdienen die besonderen Lebens- und Todesumstände des Wissenschaftlers Interesse. Dieser Beitrag liefert eine kurze Zusammenfassung seines Lebens und seiner Leistungen.

## Entwicklung

Richard Wolfgang wurde am 22. August 1859 als drittes Kind des Börsenmaklers Simon Joseph und seiner Ehefrau Henriette Semon (gesprochen Semin) in Berlin geboren [36, 54]. Die Lektüre von Charles Darwin weckte bereits auf dem Gymnasium sein Interesse an der Biologie, deren Studium er nach seinem Abitur 1879 bei Ernst Haeckel (1834 bis 1919; Arzt und Biologe) in Jena begann.

**Figure 1:**
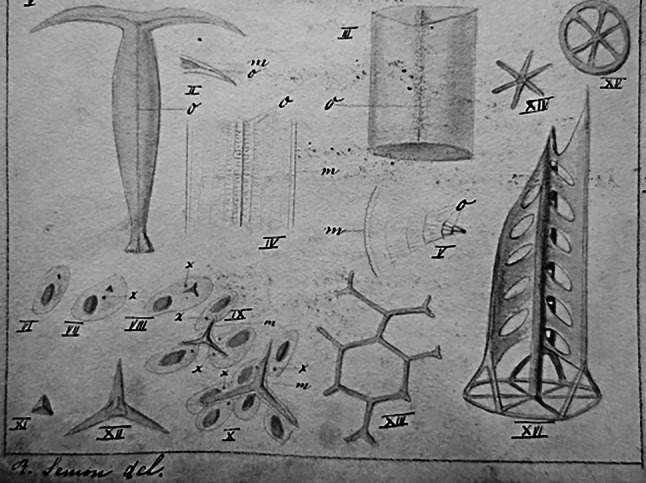

1881 nahm Semon in Heidelberg das Medizinstudium auf und betätigte sich bei dem Zoologen und Zellbiologen Otto Bütschli (1848 bis 1920). 1883 legte er in Jena seine Dissertation über die Anatomie der Seegurken (Holothurien) vor und wurde bei Ernst Haeckel zum Dr. phil promoviert (Tab. 1). 1884 bestand Semon das Medizinexamen in Heidelberg. 1885 erhielt er die Approbation und konvertierte zum Christentum. Im gleichen Jahr begleitete Semon als Arzt die Expedition des Afrikaforscher Robert Flegel (1852 bis 1886) nach Lagos, war jedoch aufgrund einer schwer verlaufenden Malaria-Erkrankung bald gezwungen nach Europa zurückzureisen und besuchte seinen älteren Bruder, den bedeutenden Hals-Nasen-Ohren Arzt Felix Semon (später „Sir Felix“) in London, ehe er im August nach Jena zurückkehrte. Mit Haeckels Unterstützung arbeitete er von Ende 1885 bis 1886 bei Anton Dohrn (1840 bis 1909) an der Zoologischen Station in Neapel und wurde 1886 Assistent bei dem Anatomen und Zoologen Oskar Hertwig (1849 bis 1922) an der Medizinischen Fakultät in Jena. Im gleichen Jahr wurde er mit einer Arbeit über den Bau und die Entwicklung kalkführender Stützgewebe im Tierreich zum Dr. med. promoviert (Abb. 1) und im Jahr danach mit seiner Studie zur indifferenten Anlage der Keimdrüsen habilitiert (Tab. 1). 1891 folgte die Ernennung zum ausserplanmässigen Professor.

**Table 1:** 

1883	Das Nervensystem der Holothurien (Zoologische Dissertation). Zeitschrift f Naturwissenschaft 16/Separatdruck
1886?	Bericht von R. Semon über die Flegelsche Lagosee-Expedition. Gedruckt, ohne Angabe zu Erscheinungsjahr, Verlag und Ort [Universitätsarchiv Jena]
1887	Beiträge zur Naturgeschichte der Synaptiden des Mittelmeeres (Inauguraldissertation der Medic. Fakultät der Universität Jena). Breitkopf & Härtel, Leipzig
1887	Die indifferente Anlage der Keimdrüsen beim Hühnchen und ihre Differenzierung zum Hoden (Habilitationsschrift). Jenaische Zeitschrift für Naturwissenschaft 22
1893–1913	Zoologische Forschungsreisen in Australien und dem Malayischen Archipel (Hrsg. & Autor). G. Fischer, Jena, 6 Bde., 112 Beiträge von insgesamt 77 Wissenschaftlern auf 5407 Seiten mit 343 Tafeln und 1810 Textabbildungen
1896	Im australischen Busch und an den Küsten des Korallenmeeres. Reiseerlebnisse und Beobachtungen eines Naturforschers in Australien, Neu-Guinea und den Molukken. Engelmann, Leipzig
1904	Die Mneme als erhaltendes Prinzip im Wechsel des organischen Geschehens. Engelmann, Leipzig
1909	Die mnemischen Empfindungen in ihren Beziehungen zu den Originalempfindungen. Erste Fortsetzung der Mneme. Engelmann, Leipzig
1912	Das Problem der Vererbung „erworbener Eigenschaften“. Engelmann, Leipzig
1920	Bewusstseinsvorgang und Gehirnprozess: eine Studie über die energetischen Korrelate der Eigenschaften der Empfindungen. Nach dem Tode des Verfassers herausgegeben von Otto Lubarsch. J. F. Bergmann, Wiesbaden

Mit Ernst Haeckels Hilfe gewann Semon die Unterstützung des Mäzens Dr. Dr. Paul von Ritter (1825 bis 1915) aus Basel für eine zweijährige Forschungsreise ([26]; [Semon 1896]; Tab. 1). Im Juni 1891 brach Semon nach Colombo, Adelaide, Melbourne, Sydney und von dort nach Brisbane auf, wo er im August eintraf. Zwischen September 1891 bis Januar 1892 führte er am Fluss Boyne ein Lagerleben im Hinterland und begann mit einer umfangreichen zoologischen Sammlung, wobei sein Hauptinteresse der Entwicklung von Lungenfischen (Ceratoden) galt. Von Februar bis April 1992 durchreiste er Queensland, von April bis Mai Neuguinea, kehrte mehrere Monate in das Camp in Queensland zurück und besuchte danach Bali und Java. Januar bis März 1893 verbrachte er in Ambon auf den Molukken. Am 1. April 1893 trat er die Heimreise über Singapur und Bombay an und traf im Mai mit sehr reichem Material im Gepäck in Jena ein.

Mäzen Paul von Ritter, Verleger Gustav Fischer und vor allem Ernst Haeckel mit seinem Kreis unterstützten die Auswertung und Publikation der Ergebnisse, die am Ende mehr als 5000 Seiten umfassten (Tab. 1). Alle Vorzeichen standen auf Erfolg bis Richard Semon Maria Krehl begegnete. Maria Krehl (1863 bis 1918), die gebildete und moderne Tochter des Verlegers Carl Geibel (1806 bis 1884), war seit 1886 mit dem bereits renommierten Pathologen Ludolf Krehl (1861 bis 1937) verheiratet und Mutter von drei kleinen Kindern. Am 27. September 1897 schrieb der Anatom und Ornithologe Max Fürbringer (1846 bis 1920) aus Jena einen Brief an den verreisten Haeckel: *… Semon steht im Begriffe etwas zu thun, was ihn für immer von Jena trennen wird: er will sich mit der Frau des ihm befreundeten hiesigen Collegen Kr. verbinden und seine hiesige Position aufgeben*. Nichts sei unversucht gelassen worden *den beiden bethörten Menschen zu helfen* [22]. Aber es handle sich um *acuten Wahnsinn* [22]. Daraufhin Haeckel an einen anderen Kollegen *… Semon mit der Frau eines Collegen (Krehl) durchgegangen!! –* [22]. – *Sehr traurige Stunden macht mir auch täglich die unglückselige Liebe-Tragödie von Prof. Semon und Frau Prof. Krehl. Die Einzelheiten dieser bösen Geschichte (die schon über ein Jahr spielen soll!) sind unbegreiflich. Sie gehen wahrscheinlich nach Australien. Schade um den ausgezeichneten Naturforscher, und um das zerstörte Glück von zwei Familien* [10]! *… Das Traurigste war aber für mich die Affaire Semon, bei der übrigens Eva *(!, hf)* Krehl die grössere Schuld trägt*…[22]. Am 09. Oktober 1897 schrieb Semon an Haeckel: *Es kostet mich ungeheure Überwindung, Ihnen nach dem, was vorgefallen ist, zu schreiben, Ihnen, der mir mehr als ein Lehrer und Freund gewesen ist, der wahrhaft väterlich für mich gesorgt hat. … Was geschehen ist, lässt sich nicht ändern, liess sich höchstens ändern, wenn wir beide gemeinschaftlich in den Tod gegangen wären. … Auf das, was man Carriere nennt, verzichte ich gern. Aber auch ohne das kann man doch noch Tüchtiges leisten. Und das verspreche ich Ihnen zu tun, soweit das irgend in meinen Kräften steht. Leben Sie wohl, mein teurer, innig geliebter Lehrer, mein väterlicher Freund* [22]. Spätestens im Sommer 1900 war Haeckel als Gast im Haus Semon ganz versöhnt und betrachtete das alles aus *„Kosmologischer Perspective“ … zumal ich an keinen freien Willen mehr glaube* [22]. Danach war der erneute vertrauensvolle Austausch in wissenschaftlichen und privaten Belangen wieder hergestellt [4].

Semons flohen nicht nach Australien, sondern nach München und heirateten 1899 [51]. Auch Ludolf Krehl verliess 1897 mit seinen Kindern Eva, Leonore und Wolfgang Jena und folgte Rufen nach Marburg, nach Greifswald und nach Straßburg ehe er 1907 Nachfolger von Wilhelm Erb in Heidelberg wurde [54]. Richard führte das Leben eines Privatgelehrten und Maria Semon war in München als Rentiére gemeldet [51]. Sie übersetzte Werke von Charles Darwin [9], Auguste Forel [15], und Lloyd Morgan [35] ins Deutsche. Maria und Richard arbeiteten und reisten. So vermerkt auch Emil Kraepelin eine Begegnung mit dem *Australienreisenden Semon im gastlichen Hause der Frau Schwingshackl *am Gardasee im Sommer 1905 [33].

## Gedächtnis und Vererbung

### Mneme-Theorie

Der Rückzug aus den unmittelbaren akademischen Verpflichtungen schuf Freiräume, die Semon nutzte um Gedanken von Ernst Haeckel in eigenen „bio-philosophischen“ Arbeiten auszubauen. Für seine Ausführungen schuf er bewusst eigene Begriffe, z. B. „Mneme“, damit sein Konzept nicht etwa mit dem engen Verständnis des umgangssprachlichen „Gedächtnis“ gleichgesetzt werde ([Semon 1904, S. 20]; Tab. 1). Mneme sei nämlich die allgemeine Eigenschaft eines Organismus Engramme zu bewahren und entsprechend angepasst auf Reize zu reagieren. Dabei entwickelten Organismen aus einem „primären Indifferenzzustand“ (1) durch die einen „engraphischen Stimulus“ eine unmittelbare, „synchrone“ engraphische Veränderung (2a) und eine etwas länger anhaltende, „akoluthe“ engraphische Veränderung (2b), die in einen „sekundären Indifferenzzustand“ übergehe (3). Auf diesen Ruhezustand des veränderten Organismus wirken alle späteren „ekphorischen“ Reize ein (4) ([Semon, 1904]; Tab. 1). Aus heutiger Sicht erscheint Semons wort- und variantenreiche Darstellung in Text und Bild mit wachsendem Umfang etwas an Schärfe zu verlieren.

In seiner nachfolgenden Arbeit über die mnemischen Empfindungen ([1909]; Tab. 1) versuchte Semon seine Theorie in zwei Grundsätzen zu formulieren. Sein erster mnemischer Hauptsatz der Engraphie lautet: alle gleichzeitigen Erregungen innerhalb eines Organismus bilden einen zusammenhängenden, simultanen Erregungskomplex, der als solcher engraphisch wirkt und damit einen ein Ganzes bildenden Engrammkomplex zurücklässt ([Semon, 1909, S. 371]; Tab. 1). Der zweite mnemische Hauptsatz der Ekphorie besagt ein simultaner Engrammkomplex bewirke die partielle Wiederkehr derjenigen energetischen Situation, die vormals engraphisch gewirkt hat. Semon bezieht sich dabei stets auf den gesamten Organismus, wenngleich er dem Gehirn durchaus eine hervorgehobene Stellung einräumt: … *die engraphische Empfänglichkeit der organischen Substanz eines Individuums (ist) zwar im Nervensystem am höchsten ausgebildet, keineswegs aber ein Monopol dieses Gewebssystems* ([Semon, 1920, S. 199]; Tab. 1). Während die Neurowissenschaft heute die Engramme im Gehirn sucht (z. B. [29, 40, 45]), dachte der Entwicklungsbiologe Semon nicht nur über das Zentralnervensystem, sondern sogar über das Individuum hinaus.

### Vererbung erworbener Eigenschaften

Der Botaniker und Zoologe Jean-Baptiste Lamarck (1744 bis 1829) hatte 1805 behauptet, alles, was im Laufe des Lebens in einem Organismus verändert werde, werde auch an die Nachfahren weitergegeben. Dies stand im diametralen Gegensatz zum Darwinschen Selektionsprinzip, vor allem in seiner engen Auslegung, die in Deutschland z. B. durch August Weismann (Tab. 2) vertreten wurde. Aber viele Forscher und Denker beschäftigten sich mit den Elementen für der Weitergabe individueller Eigenschaften, den Informationsträgern [48]: Darwin bezeichnete sie als Lebenskörperchen (Gemmulen), Haeckel als Plastidule, der Anatom und Zoologe Oskar Hertwig (1849 bis 1922) als Idioblasten. Der Schweizer Psychologe Theodule Ribot (1839 bis 1916) fasste Erblichkeit als eine spezifische Form von Gedächtnis auf: *es bedeutet für die Spezies, was Gedächtnis für das Individuum bedeutet* [46] und der Physiologe Ewald Hering (1834 bis 1918) ging noch weiter, indem er Gedächtnis für eine allgemeine Eigenschaft der organisierten Materie hielt [24].

**Table 2:** 

Name, Vorname	Geb.-verst.	Zitate	Ort: Art der Tätigkeit
Blaringhem, Louis	1878–1958	1/3	F: Botaniker, Genetiker
Bonnier, Gaston	1853–1922	2/4	F: Botaniker
Brown-Sequard, Charles Eduard	1817–1894	*4/(23)*	F & UK: Physiologe, Neurologe
Castle, William Ernest	1867–1962	2/3	USA: Zoologe, Genetiker
Cieslar, Adolf	1858–1934	1/3	(P &) A: Forstwirtschaftler, Botaniker
Cope, Edward Drinker	1840–1897	1/3	USA: Zoologe, Paläontologe
Cunningham, Joseph Thomas	1859–1935	2/3	UK: Zoologe, Meeresbiologe, Neo-Lamarckist
Darwin, Charles Robert	1809–1882	*11/3*	UK: Naturforscher
Davenport, Charles Benedict	1866–1944	3/3	USA: Biologe, Eugeniker
Fischer, Emil	1868–1954	3/7	CH: Arzt, Entomologe
Forel, Auguste	1848–1931	4/3	CH: Psychiater, Entomologe
Guthrie, Charles Claude	1880–1963	1/3	USA: Physiologe, Transplantationsmediziner
Jennings, Herbert Spencer	1868–1947	6/5	USA: Zoologe, Genetiker, Eugeniker
Johannsen, Wilhelm	1857–1927	*17/3*	DK: Botaniker, Genetiker, „Gen“
*Kammerer, Paul*	1880–1926	*34/13*	A: Vivarium, exp. Biologe
Kropotkin, Pjotr	1842–1921	2/3	R: Geograph, Zoologe, Revolutionär
MacDougal, Daniel T	1865–1958	4/10	USA: Pflanzenphysiologe, Ökologe
Merrifield, Frederick	1831–1924	2/3	UK: Anwalt, Entomologe
Morgan, Thomas Hunt	1866–1945	4/3	USA: Evolutionsbiologe, Genetiker, Nobel‑P
Osborn, Henry Fairfield	1857–1935	5/5	USA: Paläontologe, Geologe, Eugeniker
Pfeffer, Wilhelm	1845–1920	6/5	D: Botaniker
Pictet, Arnold	1869–1948	5/5	CH: Entomologe
Przibram, Hans Leo	1874–1944	*12/4*	A: Zoologe, Leiter des Vivariums Wien
Roux, Wilhelm	1850–1924	2/3	D: Anatom, Embryologe
Schübeler, Frederik Christian	1815–1892	1/3	N: Botaniker
Spencer, Herbert	1820–1903	1/3	UK: Soziologe, Evolutionstheoretiker
Standfuss, Max	1854–1917	3/6	P & CH: Theologe, Entomologe
Sumner, Francis Bertody	1874–1945	6/1	USA: Zoologe, Ichthyologe
Tornier, Gustav	1859–1938	2/3	D: Zoologe, Paläontologe
*Tower, William Lawrence*	1872–1967	*25/1*	USA: Zoologe
Weismann, August	1834–1914	*23/16*	D: Arzt, Zoologe, Genetiker, Neo-Darwinist
Woltereck, Richard	1877–1944	5/3	D: Zoologe, Hydrologe, Ökologe
u. v. a.	–	–	–

In der Spalte „Zitate“ sind angegeben die Zahl der Textseiten auf denen die Ergebnisse des jeweiligen Autors dargestellt werden/Zahl der zitierten Referenzen des Autors. Besonders viel Aufmerksamkeit schenkte Semon den Arbeiten von Kammerer und Tower (*kursiv*) ([Semon, 1912]; Tab. 1)

*A* Österreich, *CH* Schweiz, *D* Deutschland, *DK* Dänemark, *F* Frankreich, *N* Norwegen, *P* Polen, *R* Russland, *UK* Vereinigtes Königreich, *USA* Vereinigte Staaten von Nordamerika

Für Semon war die Vererbung erworbener Eigenschaften nur eine facettenreiche Fortsetzung seiner Mneme-Theorie. So beginnt er seine Ausführungen mit Beispielen, die von der ererbten Syphilis bis zur taubblinden Helen Keller und deren rascher intellektueller Entwicklung reichen; von der angeborenen zirkadianen Rhythmik im Pflanzenreich bis zu den verkümmerten Augen der Tiefseefische. Als deskriptiv und systematisch arbeitender Zoologe war Semon bei diesem Thema auch auf die Veröffentlichungen experimentell tätiger Biologen angewiesen. Gezielt begann er 1907 mit dem Zusammenstellen der verfügbaren Evidenz [31]. Dabei kam es ihm letztlich darauf an zu zeigen, dass es sich bei der Vererbung erworbener Eigenschaften nicht nur um einen Rückfall auf frühere Entwicklungsstufen (Atavismus), um Zuchtwahl (nach Mendel), das Ergebnis einer direkten Beeinflussung der Keimzellen oder gar eine Fäschung handle. In Tab. 2 ist nur ein Teil der in der *Vererbung „erworbenener Eigenschaften“* [1912, siehe Tab. 1] zitierten Autoren aufgelistet, nämlich – mit einer Ausnahme – jener, von denen Semon drei oder mehr Arbeiten berücksichtigt hat.

Zunächst fallen die Multidisziplinarität und Internationalität der verwendeten wissenschaftlichen Arbeiten auf, die zu einem erheblichen Teil nach 1900 veröffentlicht waren. Die Nähe von Genetik und Eugenik ist in der Tabelle nur andeutungsweise zu erkennen (z. B. Davenport, Kammerer, Jennings, Osborn). Durchgehend schien den Botanikern der Nachweis einer Vererbung veränderter Eigenschaften (= Pflanzenzüchtung) leichter zu fallen als den Zoologen, wenngleich damals aufgrund einiger aktueller Arbeiten gerade der Eindruck entstanden war, als würde nun auch an Tieren gelingen, was an Pflanzen bereits zuverlässig nachgewiesen worden war. Dergleichen schien sich zumindest bei den Insekten und dabei vor allem bei Schmetterlingen, etwa in den Arbeiten von Fischer und Pictet, abzuzeichnen.

Einige Autoren fallen durch die Vielzahl ihrer zitierten Studien auf oder durch die vielen Seiten, die Semon auf eine Besprechung ihrer Ergebnisse verwendete. Auch die „Gegenseite“ von Darwin selbst bis zu Richard Weismann wird umfänglich zitiert und diskutiert. In seinen Ausführungen schenkt Semon den Umwelteinflüssen auf die Individualentwicklung grosse Aufmerksamkeit, so etwa den Experimenten zur Belichtung und Pigmentierung von Plattfischen durch Cunningham, oder Kammerers Beschreibung einer fehlenden Rückbildung der Augen beim Grottenolm in heller Umgebung. Brown-Sequards Behauptungen über die Erblichkeit einer experimentell induzierten Epilepsie beim Meerschweinchen begegnet Semon mit einiger Skepsis. Für umso wichtiger hält er Kammerers Folgen einer Verstümmelung an der Schlauchseescheide (Ciona intestinalis), die in Folgegenerationen angeblich umso kräftigere Anlagen ausbildete [30].

**Figure 2:**
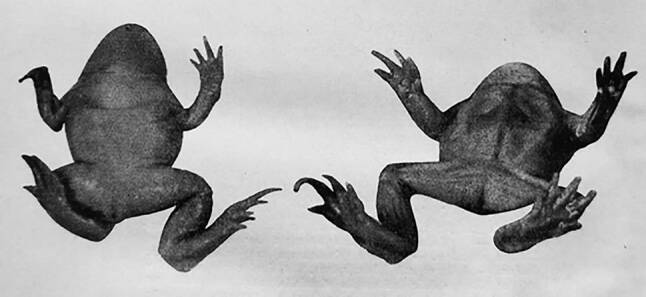

Kammerer gelang im Wiener Vivarium, einer führenden europäischen Einrichtung für experimentelle Biologie, anscheinend auch die Demonstration einer Anpassung des Feuersalamanders (Salamandra salamandra) an die Umgebungsfarbe [30]. Überragende Bedeutung erlangten seine Arbeiten an den Geburtshelferkröten (Alytes obstetricans), die sich unter Normalbedingungen an Land begatten und bei denen das Männchen die Laichschnur auf dem Rücken austrägt. Kammerer – der „Krötenküsser“ [32] – beschrieb unter anderem eine Generationen überdauernde Veränderung des Aufzuchtverhaltens wenn Alytes zur Laichablage im Wasser gezwungen wurde und ferner eine Ausbildung von Brunftschwielen über mehrere Generationen. Diese sekundären Geschlechtsmerkmale mit einer Hypertrophie der Vorderarme finden sich ansonsten nur bei Fröschen, die ihre Weibchen bei einer Befruchtung im Wasser umklammern (Abb. 2).

Ähnlich anregend waren die Untersuchungen von William L. Tower am Coloradokartoffelkäfer (Leptinotarsa decemlineata), die er 1906 auf mehr als 300 Seiten publiziert hatte [40, 53]. Ein Kernergebnis war die Veränderung von Grösse, Farbe und Zeichnung der Nachkommen wenn die Eltern während der Wachstumsperioden ihrer Keimzellen – und nur dann – experimentell veränderter Temperatur oder Feuchtigkeit ausgesetzt waren.

Semon schrieb noch kurz vor seinem Tod *… Die Möglichkeit, die Keimzellen engraphisch zu beeinflussen, ist experimentell besonders durch die Untersuchungen von Tower (1906 S. 286–294) in äusserst schlagender Weise bewiesen worden …* ([Semon, 1920, S. 200; 53]; Tab. 1). Vermutlich hatte Semon unter den Bedingungen des I. Weltkriegs nicht mehr von erheblichen Zweifeln an Towers Ergebnissen erfahren, die durch öffentlich ruchbar gewordene private Verwerfungen und einen angeblichen Brand in seinem Labor weiter geschürt wurden. Nach Towers unrühmlichem Abgang von der University of Chicago im Jahr 1917 wurde wissenschaftlich nie mehr von ihm gehört.

## Enden

### Nachrufe

**Figure 3:**
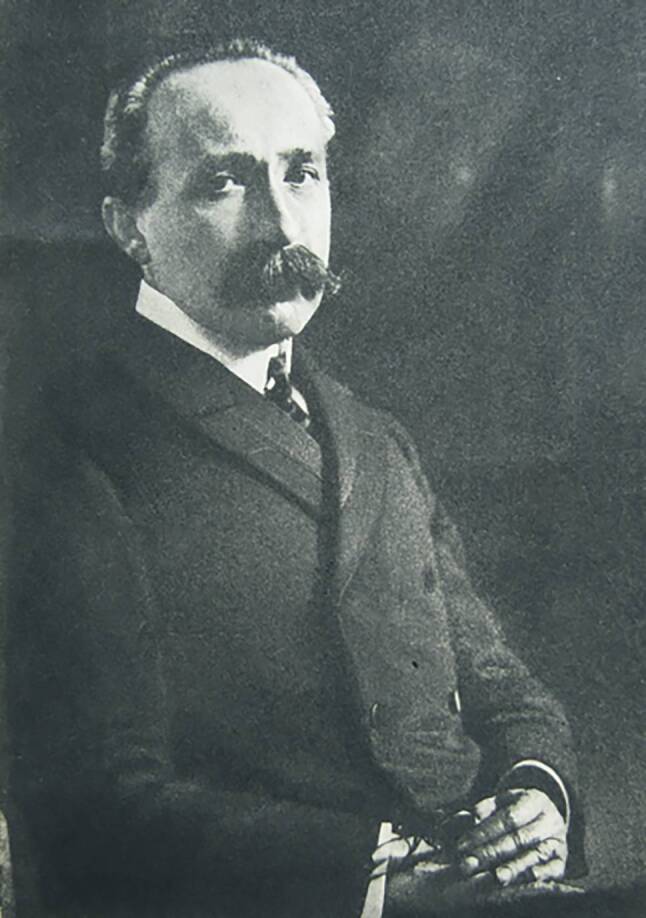

Kurz nach dem Sturz des Bayerischen Monarchen und der Ausrufung der Räterepublik in München wickelte sich Richard Semon in die Fahne des Kaiserreichs und erschoss sich im Zimmer seiner vor wenigen Monaten verstorbenen Frau [20, 48]. Das restliche Vermögen seiner Frau von 60.000 Mark hinterliessen er beziehungsweise ihre Kinder aus erster Ehe der Universität Jena für eine Stiftung. Abb. 3 zeigt ein spätes Portrait von Richard Semon.

Diesen Abschiedsbrief hatte er am gleichen Tag an Auguste Forel abgesandt [2]:

„27/XII. 18

Hohenzollernstr. 130

München

Teuerster Freund,

Der letzte Brief, den ich schreibe, ist an Sie gerichtet. Ich vermute stark, dass Sie es nicht billigen werden, wenn Sie hören, dass ich freiwillig aus dem Leben scheide. Ich würde es auch nicht getan haben und würde in der Arbeit Kraft gesucht (das habe ich) und gefunden haben, die schreckliche Vereinsamung zu ertragen, in die ich aus der höchsten geistigen Gemeinschaft heraus durch den Tod meiner unvergleichlichen Lebensgefährtin geschleudert bin. Aber die Arbeit wird unmöglich, weil das Gehirn, vor allem die Mneme in immer zunehmender Weise versagt. Bei vielen macht sich das erst mit 80 bemerkbar, bei mir schon 20 Jahre früher. Ich bin da erblich belastet. Bei meiner Mutter begann es ebenfalls sich, als sie 60 wurde, bemerklich zu machen, mit 70 war es schon sehr merklich, als sie mit 83 Jahren starb, ausgesprochene senile Demenz. Da ich vom Apfel der Erkenntnis gegessen habe, erkenne ich schon die ersten Vorzeichen, und will mein Lebenswerk nicht durch einen minderwertigen Schluss verunzieren. Ohne solche Arbeit aber kein Leben für mich. Ich habe ja auch für niemanden zu sorgen und hinterlasse keine Lücke. Also verzeihen Sie mir, indem Sie mich verstehen.

Ihnen, verehrtester Freund, habe ich für ungeheuer Vieles zu danken, ausserordentliche geistige Anregungen, tatkräftige Förderung meiner Bestrebungen und Gedanken. Ich hinterlasse mein letztes Werk: „Bewusstseinsvorgang und Gehirnprozess“ halb vollendet. Da aber der, wie ich glaube, fruchtbare Kern in den bisher vollendeten 6 ersten Kapiteln bereits deutlich erkennbar ist, habe ich Sorge getragen, dass er als Torso, der er ist, veröffentlicht wird. Ich beklage es, dass ich Ihr Urteil über dasselbe nicht mehr vernehmen kann.

Nun leben Sie wohl. Möge es Ihnen und den Ihren gut gehen. Mein Herz ist mit Verehrung und Dankbarkeit Ihnen gegenüber erfüllt.

Ihr treuer Richard Semon“

Dazu Forel in seinen Lebenserinnerungen [14, S. 347/8]: *Kurz nach dem Tod seiner lieben Frau und auch durch Deutschlands Niederlage schwer niedergedrückt, erschoss sich mein Freund Richard Semon in München. Er … bildete sich grundlos ein senil zu werden. Am Tag seines Todes schrieb er mir noch seinen letzten Brief, den ich in einem Nekrolog im „Journal für Psychologie und Neurologie“, Bd. 25, 1919 vollständig wiedergab. Der Tod Semons schmerzte mich tief; ich fühlte mich eins mit ihm in meinem wissenschaftlichen Denken; aber seine Schwermut hatte ihn über sich selbst getäuscht.* Suizide von Wissenschaftlern sind kein grosses Forschungsthema. Als Risikofaktoren gelten männliches Geschlecht, affektive Erkrankungen, Tod eines Angehörigen, (subjektive) Isolation, Ambition, schwierige politische Umstände, wirtschaftliche und rechtliche Probleme, wozu auch Zweifel an der Richtigkeit und Bedeutung ihrer Arbeit gezählt werden können [18]. Einige dieser Faktoren trafen auf Semon und nicht nur auf ihn zu.

Die rasche und vollständige Veröffentlichung eines persönlichen Abschiedsbriefes erscheint aus heutiger Sicht ungewöhnlich. Wie einem englischsprachigen Nachruf aus dem Jahr 1920 zu entnehmen ist, der ebenfalls den kompletten Brief enthält [1], gab Forel auch Details von Semons Tod in einer französischsprachigen Freidenker-Zeitschrift preis (La Libre Pensee Internationale). Der plötzliche und tragische Tod Semons wurde in einer Rezension seines letzten Buches angesprochen [8]. Auszüge aus dem Abschiedsbrief teilte auch Jelliffe, Herausgeber des Journal of Nervous and Mental Disease, in seinem ausführlichen Nachruf mit [27]. Das Beispiel von Semons Suizid lieferte den Vorspann zu umfangreichen Ausführungen über den Selbstmord im Dritten Reich [20, 27] und musste als manierierter Aufhänger zu Beiträgen über die Geschichte des modernen Gehirns herhalten: es zeige sich *in Semons Privatisierung professioneller Gegenstände* (gemeint ist das Gehirn, hf) *wie sehr das Gehirn in die lebensweltlichen Belange hineinreicht* [23]. Diese tiefe Einsicht vermag Neuropsychiater nicht zu überraschen. Weitere Quellen, die sich mit Semons Tod befassen, liessen sich zitieren. Manche Autoren betonen die Bedeutung von Semons nationaler Gesinnung und des verlorenen Krieges für seinen Suizid, da er sich in die Fahne des Kaiserreichs eingehüllt habe, ehe er sich im Zimmer seiner Frau erschoss (z. B. [20, 27]). Andere verstehen Einsamkeit und Depression als wesentliche Motive [14, 27] oder betonen, dass der Gedächtnisforscher durch ein Nachlassen seiner geistigen Leistungsfähigkeit im Mark, etymologisch „im Hirn“, getroffen war [23]. Gelegentlich wird angedeutet, er habe mit seinem Werk nicht die Resonanz erzielt, auf die er hoffte [1], da die Faszination des Haeckelschen Monismus abgeklungen sei und Semons globale Überlegungen sowie seine zitierten Belege von der strengen Sachwissenschaft nicht akzeptiert wurden [48].

Prominente Suizidopfer in der Zeit der Kriege und danach waren etwa Clara Haber (1870–1915, Berlin), Chemikerin, die sich in der Nacht als ihr Mann Fritz Haber den ersten „erfolgreichen“ Einsatz von Chlorgas feierte, das Leben nahm [17]; Victor Tausk (1879–1919, Wien), Jurist, Nervenarzt und von Freud geförderter und dann verstossener Psychoanalytiker [19]; Clemens von Pirquet (1874–1929, Wien), Allergologe und Entwickler des Tuberkulin-Tests [50]; Ludwig Haberlandt (1885–1932, Innsbruck), der die Grundlagen der Kontrazeption entwickelte und dafür herbe Kritik erfuhr [21]. Die jüdischen Neurowissenschaftler Fritz Chotzen (1871–1937, Warschau), Felix Plaut (1877–1940, Epsom), Felix Stern (1884–1941, Berlin) suizidierten sich während des III. Reiches [39] und Max Levy-Suhl (1876–1947, Amsterdam) nachdem er den II. Weltkrieg in den Niederlanden überlebt hatte [25]. Der Pathologe Theodor Fahr (1877–1945, Hamburg), der sich mit dem Nationalsozialismus recht gut arrangiert hatte, verkraftete den Verlust seines Lehrstuhls nach Kriegsende nicht [13]. Gerade ambitionierte Akademiker mit starker Zielorientierung erscheinen bei ausbleibender Gratifikation, Gegenwind, sozialer Isolation, Kontrollverlust und scheinbarer Ausweglosigkeit eine besondere Gefährdung für depressive Reaktionen und suizidale Krisen aufzuweisen [3, 16, 43]. Dies gilt auch für Ärzte [3, 12], Psychologen [34] und sogar Suizidforscher [11].

### Nachwirkungen

Rudolf Brun (1885 bis 1969), Assistent bei von Monakow und Forel, später als Neurologe und Analytiker tätig, sah seine kritisch-experimentelle Studie über die Raumorientierung der Ameisen im Jahr 1904 als Beitrag zu Semons Theorie der Mneme [7]. Erwin Schrödinger schreibt in seinen Lebenserinnerungen, er sei Anfang 1918 tief in die *Schriften von Spinoza, Schopenhauer, Mach, Richard Semon und Richard Avenarius* versunken gewesen [49]. Bertrand Russell vertritt 1921 in „Analysis of Mind“ [44, 47] ein ähnlich umfassendes Gedächtniskonzept von Anpassung über Instinkt bis zur gezielten Reaktion wie Semon und bezieht sich dabei stets auf ihn. Kapitel IV über den Einfluss der Erfahrung auf das gegenwärtige Leben eines Organismus besteht in einer Verarbeitung von Semons Mneme [1904] und seiner Mnemischen Empfindungen [1909]. Smith Ely Jelliffe stellte in seinen Ausführungen zur Paläopsychologie [28] eine Verbindung zwischen Semons Mneme und dem kollektiven Unbewussten her. Eugen Bleuler übernahm Semons Terminologie und Konzepte in der Naturgeschichte der Seele [5, 6] und zahlreichen anderen Schriften [41]. Der Begriff der Mneme wirkt – vermittelt durch Maurice Maeterlinck [37] – bis in Richard Dawkins’ „Meme“-Theorie fort [10], der Ansteckung mit Ideen.

### Nachtrag

Acht Jahre nach Semons Suizid erschoss sich Paul Kammerer in der Nähe von Wien, neben Tower [53] Semons wichtigster Kronzeuge für die Vererbung erworbener Eigenschaften [30, 32, 52]. Kammerers legendäre Geburtshelferkröten wurden Opfer der Entbehrungen des I. Weltkriegs und nur ein einziges, möglicherweise manipuliertes Exemplar mit recht dubiosen Brunftschwielen war asserviert worden (Abb. 2). Die Zweifel an den Ergebnissen des begabten Experimentators und sendungsbewussten Selbstdarstellers wurden immer lauter und auch in wissenschaftlichen Journalen vorgetragen [42, 52, 55]. Seine geliebte Laborantin Alma Mahler [38] und einige Kollegen hatten den Fortgang seiner Experimente genauer registriert als er selbst. Nachdem sich in Wien keine geeignete akademische Position aufgetan hatte, berief ihn Anatoli Lunatscharsky, der sowjetische Volkskommissar für Aufklärung, 1926 als Gründungsdirektor eines neuen Moskauer Forschungsinstituts [38, 52]. Kammerer packte seine Sachen für den Umzug, schrieb aber am Tag vor seinem Suizid einen Brief aus Wien an das Präsidium der Kommunistischen Akademie [31]: *… obwohl ich selbst an diesen Fälschungen des Belegexemplars unbeteiligt bin, *(darf ich mich)* nicht mehr als den geeigneten Mann ansehen, Ihre Berufung anzunehmen. Ich sehe mich aber auch ausserstande, diese Vereitlung meiner Lebensarbeit zu ertragen, und hoffentlich werde ich Mut und Kraft aufbringen, meinem verfehlten Leben morgen ein Ende zu bereiten.*

Dieser Brief fand über Prawda und New York Times seinen Weg in die Lokalpresse und in Science [31]. Die Causa Kammerer diente zeitweise als Munition in der teilweise dramatisch geführten ideologischen Auseinandersetzung zwischen Sowjetkommunismus versus westlichen Kapitalismus; Neo-Lamarckismus mit dem Ziel der Erzeugung neuer, besserer Menschen versus Neodarwinismus mit Selektion und Eugenik [32, 52].
